# Retrospective study of canine leishmaniosis in Israel

**DOI:** 10.1186/s13071-025-06862-4

**Published:** 2025-07-04

**Authors:** Gad Baneth, Michal Peri Markovich, Yaarit Nachum-Biala, Daniel Yasur-Landau, Monica Leszkowicz Mazuz, Charles L. Jaffe

**Affiliations:** 1 Koret School of Veterinary Medicine, The Hebrew University, Rehovot, Israel; 2 Israeli Veterinary Services, Bet Dagan, Israel; 3 Division of Parasitology, Kimron Veterinary Institute, Bet Dagan, Israel; 4 Department Microbiology and Molecular Genetics, The Hebrew University - Hadassah Medical School, Jerusalem, Israel

**Keywords:** Middle East, Zoonosis, Dog, Sand fly

## Abstract

**Background:**

Canine and human leishmaniasis are prevalent around the Mediterranean Basin, where dogs are considered to be the main peridomestic reservoir for human disease. Canine leishmaniosis was initially diagnosed in central Israel in 1994. Since then, it has been routinely detected in the canine population of Israel, while only a very small number of human cases are reported annually.

**Methods:**

This retrospective analysis of dogs diagnosed with *Leishmania* infection at the Hebrew University from 1994 to December 2023 included 658 dogs.

**Results:**

Although infected dogs were reported in all 16 subdistricts of Israel, the geographic distribution and locations of infected dogs focused mainly on central and northern Israel. From 2020, isolated cases have also been detected in southern Israel, suggesting a southward spread of disease. A majority of infected dogs (*n* = 341, 51.8%) were residents of rural localities, with the remainder (*n* = 267, 40.6%) present in urban areas. The highest number of cases was found in the Jerusalem Subdistrict, but the highest density of infected dogs, cases per square km, was present in the Tel Aviv Subdistrict. The incidence rate of clinical leishmaniosis for the years 2007–2023, based on the Israeli dog registry, ranged from 0.03 to 0.09 per 1000 registered dogs. Of the 353 dogs diagnosed from 2013 onward, age ranged from 3 months to 14.5 years with a median of 4.5 years. Of the 38 dogs who were also tested by polymerase chain reaction (PCR) for *Leishmania* spp. since 2010, 32 (84%) were infected by *L. infantum*, 4 (11%) with *L. tropica*, and 2 (5%) with *L. major*.

**Conclusions:**

The epidemiology of canine leishmaniosis caused mostly by *L. infantum* presents a contrary picture to that of human leishmaniasis in Israel, in which more than 99% of patients are reported to have cutaneous leishmaniasis caused mostly by *L. major* and *L. tropica,* and less than 1% have visceral disease due to *L. infantum*. The widespread presence of canine *L. infantum* infection in central and northern Israel constitutes a zoonotic reservoir for disease for humans, wildlife, cats, and other dogs.

**Graphical Abstract:**

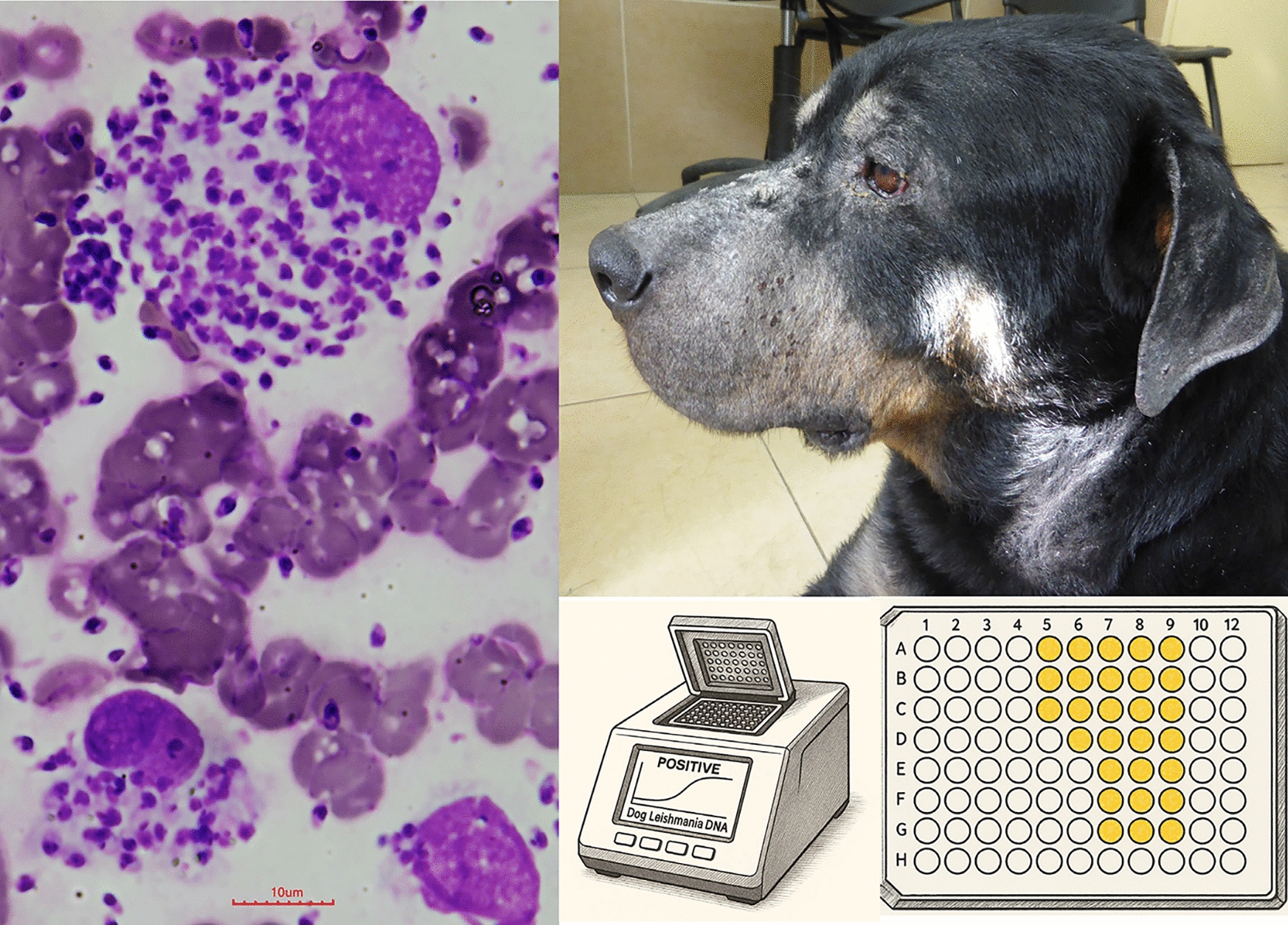

**Supplementary Information:**

The online version contains supplementary material available at 10.1186/s13071-025-06862-4.

## Background

Leishmaniasis is a group of diseases caused by kinetoplastid protozoa transmitted by sand flies and is widespread in some areas of Asia, Europe, Africa, and the Americas [1]. Leishmaniasis is endemic in Israel where three main *Leishmania* species cause disease in humans: *Leishmania major* and *Leishmania tropica* responsible for cutaneous leishmaniasis, locally termed as the Rose of Jericho; and *Leishmania infantum*, responsible mainly for visceral leishmaniasis [2]. Transmission of *Leishmania donovani* has been reported to occur in southern Israel and is associated with rare cases of human cutaneous leishmaniasis [3]. Dogs with clinical disease due to leishmaniosis are infected mainly by *L. infantum,* and rarely by *L. tropica* and *L. major* [4, 5].

The aim of this study was to retrospectively summarize the cases of canine leishmaniosis diagnosed at the vector-borne disease research laboratory (VBDRL) of the Koret School of Veterinary Medicine at the Hebrew University in Israel during 1994–2023, and the epidemiological trends of this infection. The VBDRL is the main laboratory that performs serological diagnosis of animal leishmaniosis in Israel and the only laboratory in Israel that carries out quantitative serology for canine leishmaniosis.

## Methods

### Animal samples

Serum samples of seropositive dogs submitted owing to suspicion of leishmaniosis to the VBDRL at the Koret School of Veterinary Medicine, Hebrew University, in Rehovot, Israel, during January 1994 to December 2023, were included in the study. Data on each animal included: dog name, owner name, location, date of diagnosis, sex, and age.

Some of the dogs tested serologically at the VBDRL were also tested by PCR of blood, lymph node, skin, or other tissues. This was of particular importance when suspicion of infection with *Leishmania* spp. other than *L. infantum* was present, as a small part of the infected dogs were diagnosed with infection of *L. tropica* or *L. major*.

### ELISA serology

Antileishmanial antibodies were determined by enzyme-linked immunosorbent assay (ELISA) with crude leishmanial antigen [4]. Dog serum samples were diluted to 1:100 in 96-well plastic plates and incubated with leishmanial antigen (*L. infantum* strain MCAN/IL/2010/TR1) for 1 h at 37 °C. The plates were then washed three times with 200 μl 0.1% Tween-20 in 50 mM phosphate-buffered saline (PBS) at pH 7.2. Sera were incubated with Protein A conjugated to horseradish peroxidase (Zymed Inc., San Francisco, CA, USA) at 1:10,000 dilution at 37 °C for 1 h. Unbound conjugate was washed away three times with PBS-Tween, and the plates were developed by adding the substrate 2,2′-azino-di-3-ethylbenzthiazoline sulfonate (ABTS; Sigma-Aldrich-Merck, Jerusalem, Israel). Plates were read when the absorbance (lambda of 405 nm) of the positive reference serum reached an optical density (OD) value of 1.2–1.4. Negative and positive reference dog sera were included on each plate to monitor interassay variation. A serological cutoff value of 0.4 OD was calculated on the basis of four standard deviations above the mean OD values of readings from eight control sera of PCR-negative dogs from nonendemic areas for leishmaniosis.

### PCR

PCR was performed for some of the dogs when infection with *Leishmania* spp. needed additional verification other than serology and when there was suspicion of infection by a *Leishmania* sp. other than *L. infantum* on the basis of the clinical presentation of the dog. DNA was extracted from 200 µL of ethylenediaminetetraacetic acid (EDTA)-anticoagulated blood or from tissue samples using the Illustra blood genomicPrep Mini Spin Kit (GE Health care, Buckinghamshire, UK), according to the manufacturer’s instructions. *Leishmania* detection was carried out by PCR using primers ITS-219F and ITS-219R to amplify a 265-bp fragment of the *Leishmania* ribosomal operon internal transcribed spacer 1 (ITS1) region and then evaluated by ITS1 high resolution melt (HRM) analysis (*Leishmania* ITS1 HRM PCR) [6]. PCR was performed using the StepOnePlus real-time PCR thermal cycler (Applied Biosystems, Foster City, CA, USA) as previously described [7]. The *Leishmania* ITS1 HRM PCR was performed in a volume of 20 μl containing 4 μl DNA, 400 nM of each primer, 10 μl Maxima Hot Start PCR Master Mix (2×) (Thermo Scientific, Epsom, Surrey, UK), 50 μM of SYTO9 solution (Invitrogen, Carlsbad, CA, USA), and DNase/RNase-free sterile water (Sigma, St. Louis, MO, USA). Initial denaturation for 5 min at 95 °C was followed by 45 cycles of denaturation at 95 °C for 5 s, annealing and extension at 60 °C for 20 s, and final extension at 72 °C for 10 s. Amplicons were subsequently subjected to a HRM step with the temperature raised to 95 °C for 10 s and then lowered to 60 °C for 1 min. The temperature was then raised to 95 °C at a rate of 0.3 °C per second. Amplification and HRM profiles were analyzed using the StepOnePlus series. DNA extracted from promastigotes of *L. infantum* was used as positive control for PCR and DNA from colony-bred dogs negative by PCR for vector-borne pathogens was used as a negative control. A nontemplate control (NTC) with the same reagents described above but without DNA was added to each PCR to rule out contamination. Samples were tested in duplicates.

Positive *Leishmania* ITS1 HRM PCR amplicons were sequenced at the Center for Genomic Analyses at the Hebrew University (Jerusalem, Israel) using the Big Dye Terminator cycle from Applied Biosystems ABI3700 DNA Analyzer. The ABI Data Collection and Sequence Analysis software (ABI, Carlsbad, CA, USA) was used for analysis. DNA sequences were compared with other sequences deposited in GenBank using the BLASTn website hosted by NCBI, National Institutes of Health, USA [8].

### Retrospective analysis of data

Dogs were analyzed by sex, age, and geographic location. Starting in 2013, sex and age were retrieved from each animal’s medical records. Percentage of males and females was calculated, and the age distribution evaluated. The number of dogs registered at the Israeli Ministry of Agriculture between 2007 and 2023 was retrieved from the annual reports of the Israeli Veterinary Services [9]. The yearly dog population was used as the denominator for the calculation of yearly incidence. The trend in disease incidence over time was evaluated using a Poisson regression model adjusted for population size (via an offset). To evaluate the sex distribution, the cases yearly sex distribution (in years for which sex data completeness was 80% or above) was compared with the yearly general population sex distribution using Chi-squared tests. To control for type I error due to multiple comparisons, *P* values were adjusted using the Bonferroni correction method.

The percentage of cases since 2010 was calculated for 15 cities where data on dog populations were available, and compared with their proportion in the general population. Each dog’s residence was classified as either rural (< 2000 residents) or urban (> 2000 residents) and then matched with the appropriate geographic subdistrict according to the Israel Central Bureau of Statistics (CBS). Percentage of cases per rural or urban locality was calculated. Each dog’s residency was geocoded and converted into a spatial dataset using the computer package sf (R Foundation^®^) [10–12]. Case density was calculated using the st_intersects s and st_area functions in the sf package. Maps were created with ggplot2 using R software version 4.3.1 (R Foundation^®^) [13]. A kernel density estimation (KDE) was performed to assess the spatial distribution of reported disease cases using the Terrestrial Environment Regional Analysis (Terra^®^) software package in R. A raster template was created at a spatial resolution of 1000 m. Smoothing was applied using a Gaussian kernel with a sigma of 500 m and a nine-cell window. The resulting KDE raster was normalized from 0 to 1 to represent relative case density, where 1 indicates the highest estimated intensity of cases.

## Results

**Fig. 1:**
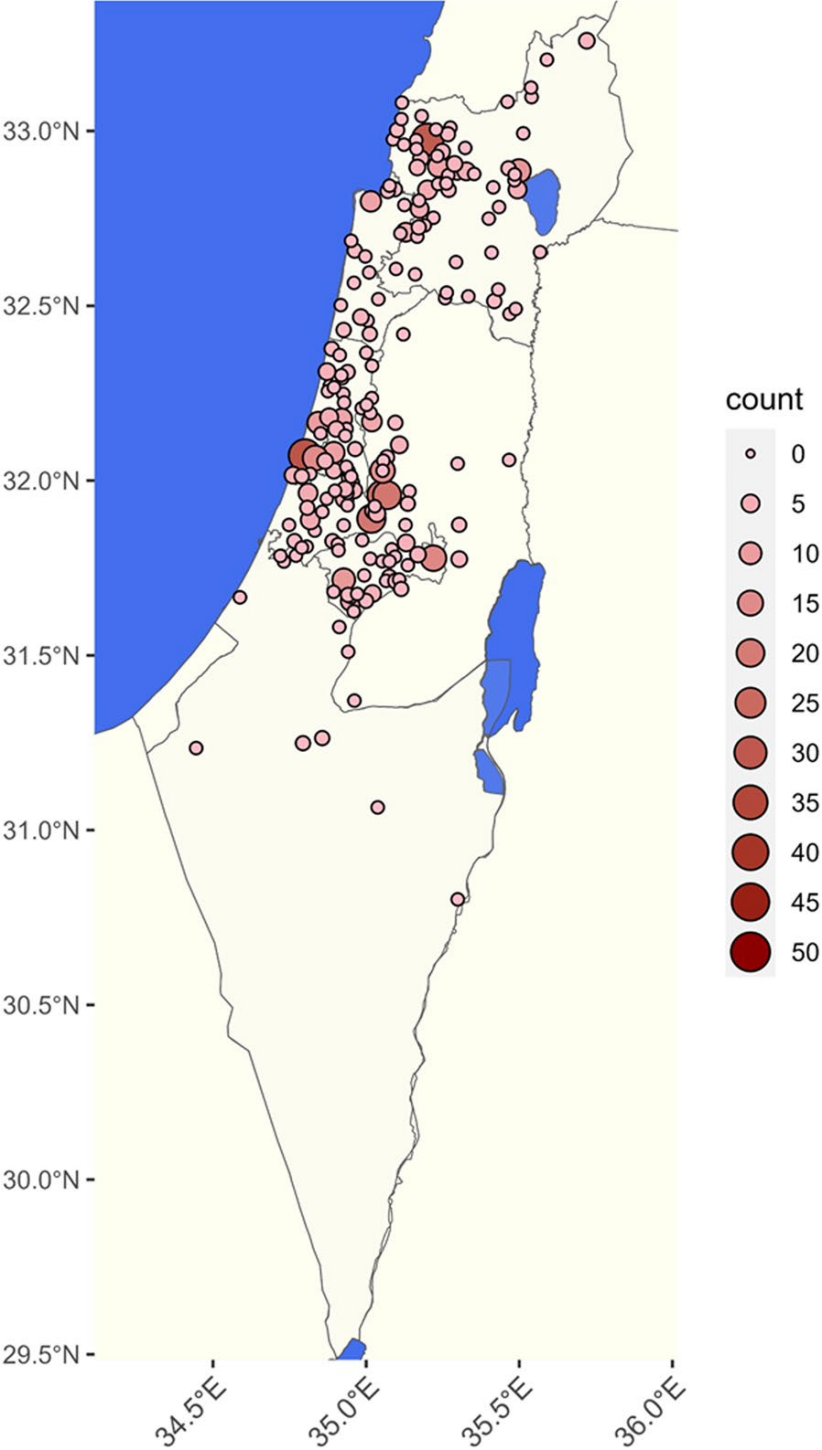
Geographic distribution of dogs diagnosed with *Leishmania* spp. infection from 1994 to 2023 with color gradient for the number of cases found in each location

**Fig. 2:**
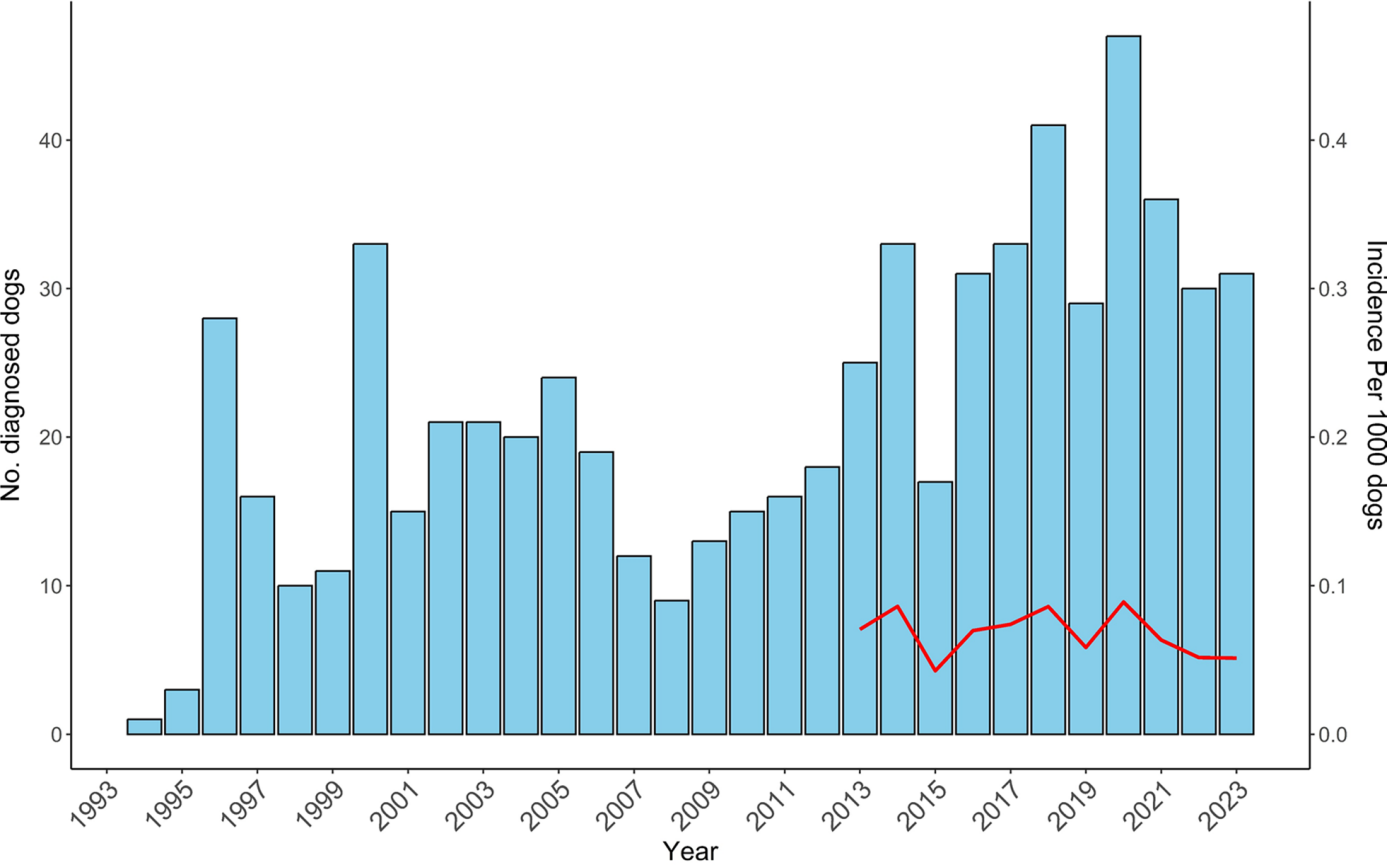
The annual number of dogs diagnosed as seropositive for *Leishmania* spp. infection from 1994 to 2023. Annual incidence beginning in 2013 is marked with a red line

**Fig. 3:**
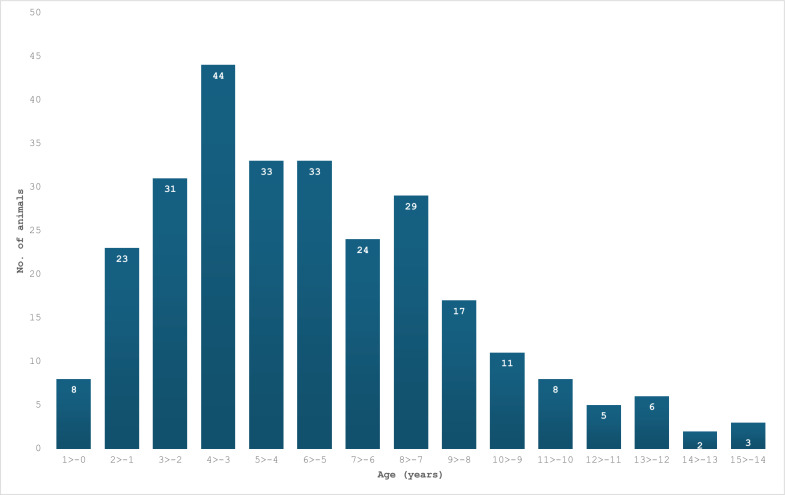
The ages of dogs diagnosed as seropositive for leishmaniosis from 2014 to 2023. The number of dogs per year of age is denoted on the top of the bar

A total of 658 dogs were diagnosed with leishmaniosis during 1994–2023. The geographic distribution of infected dogs ranged across central and northern Israel (Fig. 1). The number of dogs diagnosed with leishmaniosis per year ranged from 1 case in 1994 to 47 in 2020 (Fig. 2). The number of dogs registered during 2007–2022 at the Israeli Ministry of Agriculture increased from 346,878 to 581,022, an increase of 67.5% (Additional file 1: Additional Table 1). The cumulative incidence for the years 2007–2023 based on the Israeli dog registry ranged from 0.03 to 0.09 per 1000 registered dogs. Poisson regression model adjusted for population size showed no statistically significant increase in incidence over time (estimate −0.024, *P* = 0.161). Information regarding age was available for 277 dogs. Age ranged from 3 months to 14.5 years with a median of 4.5 years [interquartile ratio (IQR) 3–7] (Fig. 3).

Out of 353 dogs diagnosed from 2013 onward, data regarding sex was available for 278 (78.8%) dogs, of which 116 (41.7%) were females and 162 (58.3%) males. The yearly male to female ratio among dogs with known sex is presented in an additional file (Additional file 2: Additional Fig. 1). In the years 2016, 2017, and 2021–2023, sex data were available for at least 80% of reported cases. For these years, the distribution of sex among cases was compared with that of the general population using Chi-squared tests. The unadjusted *P*-values for the 5 years were 0.491, 0.948, 0.773, 1.000, and 0.655 (all with one degree of freedom). After applying the Bonferroni correction for multiple comparisons, all adjusted *P* values were equal to 1.000 (Fig. 3).

**Fig. 4:**
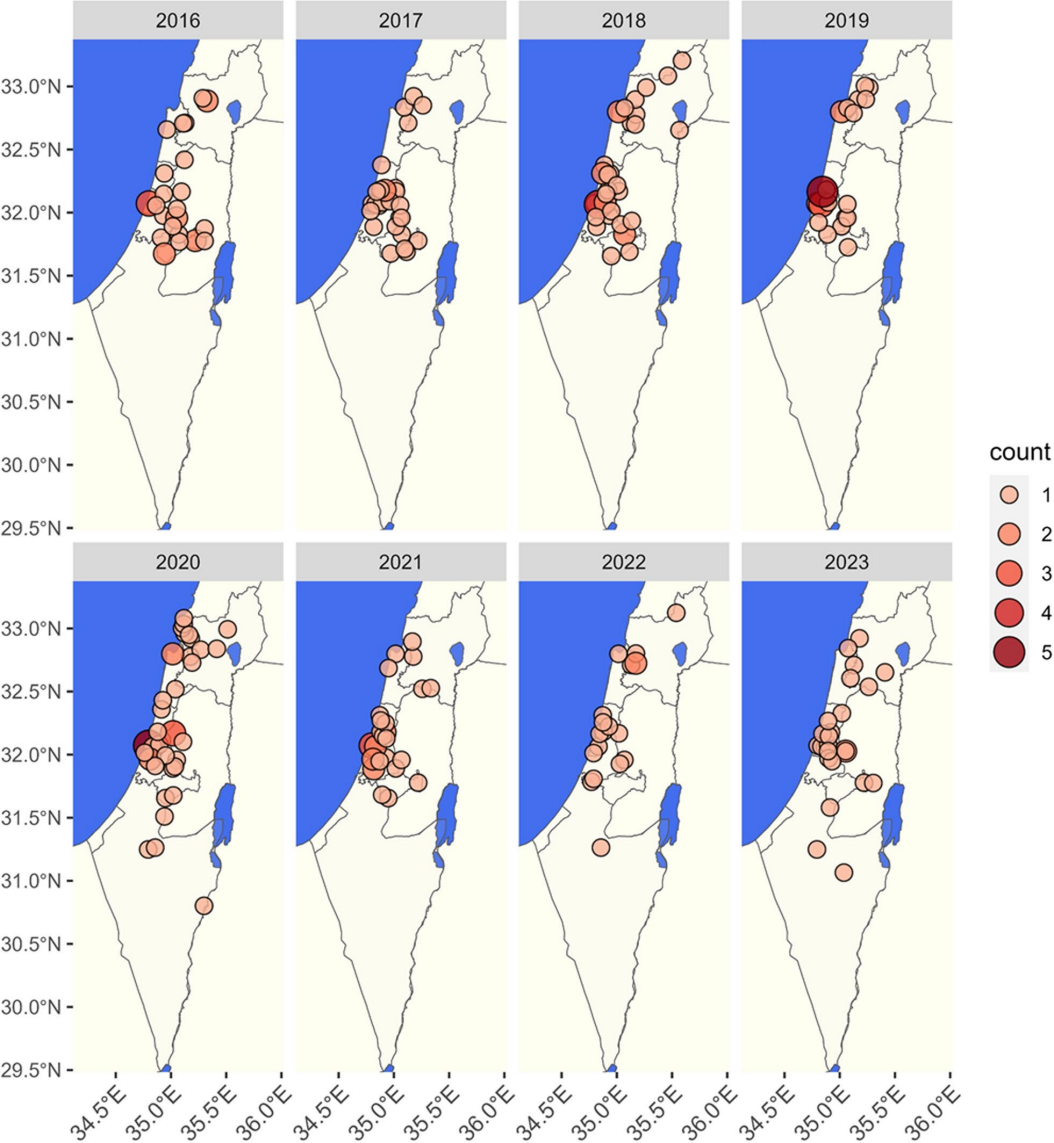
The geographic distribution of dogs detected as positive for *Leishmania* spp. in Israel shown for each year from 2016 to 2023

**Fig. 5:**
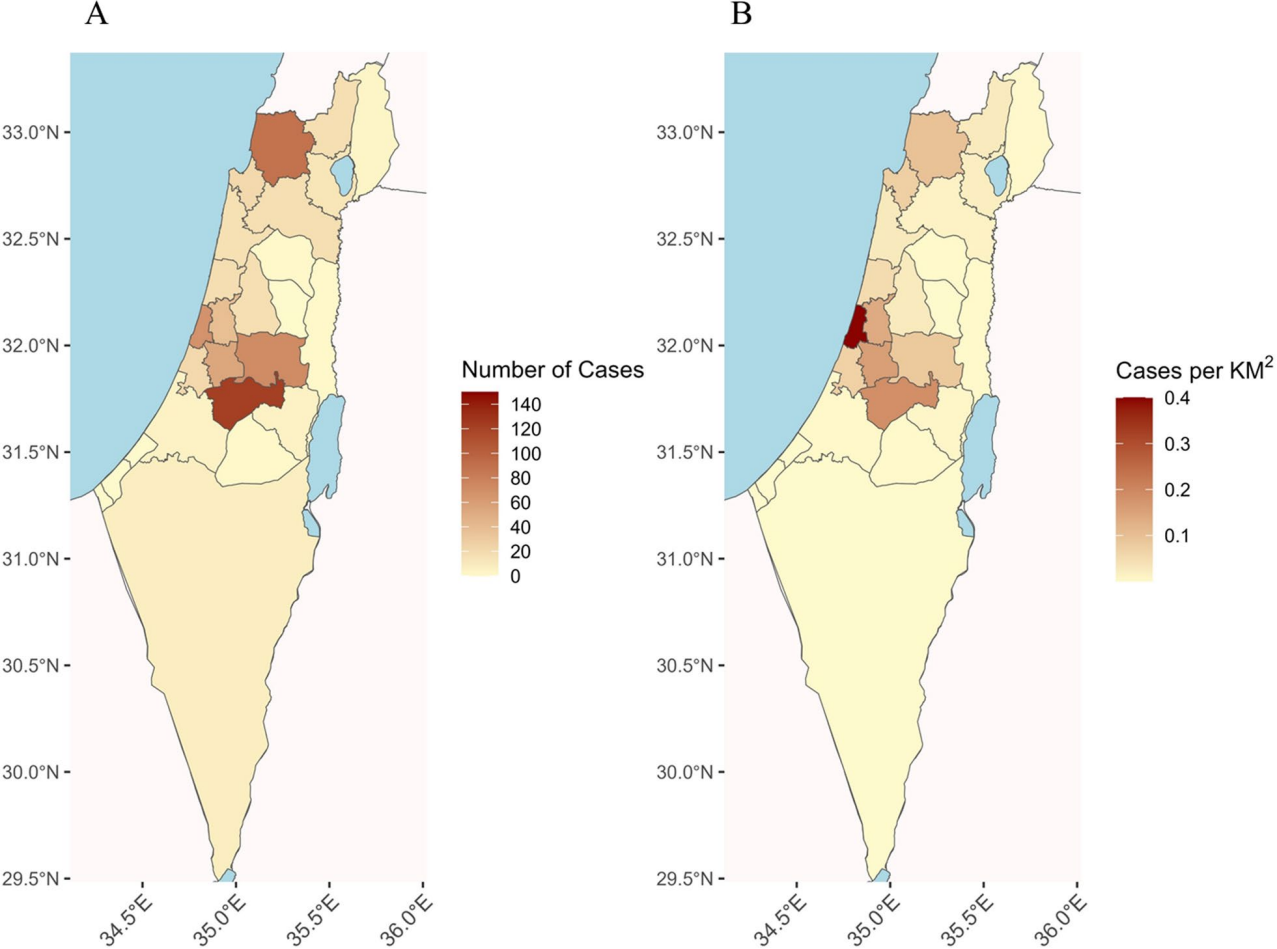
Map of Israel depicting the number of canine leishmaniosis seropositive dogs in each subdistrict (**A**) and per square kilometer (**B**)

Data regarding precise geographic location were available for 608 dogs (92.4%). Information only on the subdistrict of habitation was available for an additional eight dogs. The geographic range where infected dogs were found extended from northern Israel to the Arava and Negev deserts in the south, although a majority of animals were from central and northern Israel (Figs. 1 and 4). No cases were reported in southern Israel before 2019. However, after this time, between 2020 and 2023, infected dogs were detected in the Negev Region of southern Israel, suggesting a southward spread of disease (Fig. 4). Three hundred forty-one (51.8%) of the dogs were residents of localities regarded as rural and 267 (40.6%) of urban localities. According to the Israeli dog registry for the year 2020 [14], 38.9% of the dogs registered in Israel were residents of 15 large cities. Altogether, 27.4% (95%CI 23.2–32.0%) of the infected dogs during this study’s time period were residents of these large cities. Infected dogs were reported in all 16 subdistricts of Israel as defined by the Israeli CBS (Table 1). The highest number of cases was found in the Jerusalem Subdistrict (Fig. 5A), yet the highest density of cases per square km was found in the Tel Aviv Subdistrict (Fig. 5B). The KDE analysis allowed further visualization of the spatial distribution with hotspots of infection showing three hotspots in central Israel, near the cities of Tel Aviv, Jerusalem, and Modiin, and one in northwestern Israel near Acre (Additional file 3: Additional Fig. 2).

**Table 1:** Distribution of dogs seropositive for *Leishmania* sp. from 1994 to 2023 by subdistrict in Israel

Subdistrict	Number of dogs	Percentage of total dog number
Jerusalem	128	20.8
Judea and Samaria	98	15.9
Acre	86	14.0
Tel Aviv	69	11.2
Ramla	54	8.8
Petah Tikva	39	6.3
Rehovot	23	3.7
Haifa	21	3.4
Jezreel	20	3.3
HaSharon	18	2.9
Safed	17	2.8
Hadera	16	2.6
Kinneret	13	2.1
Beersheba	7	1.1
Ashkelon	4	0.7
Golan	3	0.5
Total	616	100

Of the 38 dogs tested by PCR for *Leishmania* spp. since 2010, 32 (84%) were infected by *L. infantum*, 4 (11%) by *L. tropica*, and 2 (5%) by *L. major* (Additional file 4: Additional Table 2). The dogs infected with *L. major* were a 5-month-old female from the Jerusalem area diagnosed in 2015 and a 7-month-old male from Jerusalem diagnosed in 2016. Both dogs had skin lesions and were described in previously published case reports [5, 15]. Dogs infected with *L. tropica* included a 1-year-old male from Ma’ale Adumim diagnosed in 2013 and reported in a previous case report (this city is a documented focus of human cutaneous leishmaniasis caused by *L. tropica*, located east of Jerusalem [16]); a female puppy of unknown age from Tel Aviv diagnosed in 2015 [5]; a 3-year-old female from Tivon near Haifa diagnosed in 2018; and a 7-month-old male from the Arava desert in southern Israel diagnosed in 2021. All the *L. tropica*-infected dogs had skin lesions.

## Discussion

The Mediterranean Basin and Middle East are endemic for leishmaniosis, and Israel, situated at the eastern edge of the Mediterranean Sea, is at the crossroads of the Middle East and the Mediterranean regions. Israel is endemic for both human cutaneous and visceral leishmaniasis, with abundant populations of sand fly vectors characteristic of the Eastern Mediterranean phlebotomine fauna [2, 17]. The first known case of human visceral leishmaniasis in Israel was reported in 1930 from a 4-year-old boy at Kibbutz Ein Harod in the Jezreel Valley, northern Israel. At the time, this area was part of the British colonial mandate [18]. Visceral leishmaniasis in humans and dogs was further identified in northern Israel in the 1930s and was subsequently reported in humans in the 1940s in the Haifa, Nablus, Jaffa, and Jerusalem districts [2]. During 1950–1992, human visceral leishmaniasis disappeared from central Israel and remained limited to a focus in northern Israel, where occasional cases of disease were reported and epidemiological studies revealed evidence of subclinical infection [19–21]. Canine leishmaniosis was rarely reported in Israel during that period, except for a survey in 1988 in Wadi Hamam near Tiberias in northern Israel, where 3 of 75 (4%) dogs were seropositive, and the parasite *L. donovani* sensu lato, probably *L. infantum*, was isolated in culture from one dog [22]. The first case of canine leishmaniosis in central Israel was diagnosed in the Village of Nataf, 12 km west of Jerusalem, in 1994 [23]. Since then, an increasing number of canine cases of leishmaniosis caused by *L. infantum* have been reported from central and northern Israel [4, 24, 25].

This retrospective analysis of the canine leishmaniosis cases diagnosed at the VBDRL over 29 years presents important information regarding the location of infected dogs in Israel and the spread of infection over time. Although most cases of canine leishmaniosis during the survey period were diagnosed at the Hebrew University VBDRL, the only laboratory in Israel that provides quantitative serology for this infection, some additional cases which were not included in the study were diagnosed by serology kits, cytology, histopathology, or PCR in veterinary clinics and other laboratories. Considering that dogs are the main peridomestic reservoirs for human infection [26], the prevalence of the canine infection may indicate sites where human disease can occur. In the current study, canine leishmaniosis was found to be distributed throughout central and northern Israel, with the highest number of cases as well as cases per km^2^ found in central Israel close to Jerusalem and Tel Aviv. A high number of cases was also present in the Acre Subdistrict, northwestern Israel. Only a few seropositive dogs were found in south-central Israel near the City of Beersheba and further south. The distribution, number, and density of seropositive dogs partially parallels the major human population centers in Israel where more owned dogs are present. However, Beersheba, the fourth largest city in Israel, and the district surrounding this city, had a very low number of dog cases. This finding challenges the assumption that infected dog distribution strictly reflects overall dog population distribution. According to a report from the Israeli Ministry of Agriculture in 2020, Beersheba had the fourth largest dog population in Israel with 16,013 registered dogs, following Haifa with 16,585, Rishon Lezion with 17,270, and Tel Aviv with 39,373 dogs [14]. Despite this, the number of canine leishmaniosis cases in the Beersheba Subdistrict was lower than in the three districts mentioned above, with only 7 cases recorded between 1994 and 2023. This accounted for 1.06% of the total cases recorded during this time period (Table 1). The Beersheba Subdistrict is the largest in Israel, extending from the south center of the country to Eilat located on the Red Sea at the country’s southernmost tip (Fig. 5). Thus, the small number of canine leishmaniosis cases detected in this region is even more meaningful when considering that it encompasses about a half of Israel’s area.

The location of human patients with visceral leishmaniasis in Israel, as described in previously published studies, shows that, between 1960 and 2000, human cases were detected only in northern and central Israel [21]. A later study on human cutaneous leishmaniasis caused by *L. infantum* covering the period from 2018 to 2021 reported five human cases in central Israel, as well as two cases in southern Israel [27].

Although the number of dogs diagnosed annually increased from only individual animals in the 90s to presently several dozen per annum, the calculated incidence rates during the years 2007–2023 did not show a parallel increase. The Israeli dog registry was used as a denominator for calculation of the disease rate. This lack of increase in incidence could be because disease incidence increased during earlier years and reached a plateau before 2007. Alternatively, past insufficient public and veterinary awareness of canine leishmaniosis and limited accessibility to diagnostic testing may have led to the increase in the number of cases between 1994 and 2007.

When analyzing the age of dogs diagnosed with leishmaniosis from 2014 to 2023, the median age was 4.5 years and the age for which the highest number of diagnoses were made was 3 years old. The numbers diagnosed at the ages of 4–8 years remained high and then decreased steadily (Fig. 3). These findings are in agreement with a study on 390 seropositive dogs with clinical leishmaniosis treated at the Autonomous University of Barcelona (UAB) veterinary hospital in Spain from 1998 to 2002, in which the peak of disease was in 2–4-year-old dogs. In addition, the study from the UAB also detected a second but lower peak at 7 years, which was not found in the current study. After this age, there was a decline in the number of dogs with disease [28]. The youngest dog diagnosed with the disease in the UAB study was 6 months old while the oldest was 13 years old, somewhat different from our findings with a 3-month-old dog infected from Israel. In addition, the UAB study found a majority of male dogs with leishmaniosis (61%) compared with females (39%). The percentage of males in the reference population of all dogs referred to the UAB veterinary hospital was only 53%. Our study found that ~58% of the dogs diagnosed with leishmaniosis at the VBDRL during 2014–2023 were males and the yearly female–male ratio was similar to the ratio in the general dog population in the selected years.

Subclinical infection is very common in canine leishmaniosis and has been shown to be considerably more prevalent than clinical disease in dogs living in endemic areas [29, 30]. The present study focused on dogs with suspected clinical disease due to *Leishmania* spp. An additional serological and molecular survey of subclinical infections in dog populations in Israel, ongoing in our laboratory, will expand our knowledge and shed light on these silent infections in the general dog population.

Although dogs with clinical infections caused by *L. tropica* or *L. major* have been described in Israel, our study indicates that these two species only caused a small percentage, as compared with *L. infantum*, of the total canine leishmaniosis infections in Israel. PCR results from 38 dogs included in the study showed that a majority of these dogs (84%) were infected with *L. infantum*. This finding supports the premise that *L. infantum* is even more prevalent among dogs with leishmaniosis who were tested serologically, as PCR and DNA sequencing were carried out only when suspicion existed that the infection was due to species other than *L. infantum*, e.g., atypical lesions including proliferative mucocutaneous lesions, dogs from highly endemic regions for human cutaneous leishmaniosis, dogs from a nontypical area for canine leishmaniosis such as the Arava desert in southern Israel, or very young dogs with skin lesions. Antibodies against other *Leishmania* spp. cross react with crude *L. infantum* promastigote antigen, therefore ELISA using this antigen may give positive reactions when sera from dogs infected with other *Leishmania* spp., such as *L. major* and *L. tropica*, are used [5]. However, the vast majority of infected dogs in Israel and the neighboring Palestinian authority tested by PCR and/or DNA sequencing have been infected with *L. infantum* [7, 25, 31], thus it can be assumed, on the basis of previous studies and the current study, that clinical infection in dogs with *L. major* and *L. tropica* is marginal. The ages of dogs included in this study with *L. tropica* or *L. major* infection tended to be young; 5/6 dogs were ≤ 1 year old. The remaining dog was 3 years old. This differed from the majority of other dogs with leishmaniosis, which belonged to older age groups.

Antibodies to *Trypanosoma* spp. could be cross-reactive with leishmaniosis in dogs, as has been shown with canine *Trypanosoma cruzi* and *Trypanosoma caninum* in South America [32–34]. Although some camel and equine cases of *Trypanosoma evansi* have been reported from southern Israel [35], no reports of autochthonous canine infection with a *Trypanosoma* sp. have been published so far, and no serological cross-reactivity between canine *Leishmania* spp. and *T. evansi* has been reported to our knowledge. Thus, it is unlikely that dogs diagnosed with *Leishmania* infection and included in the current study were seropositive due to trypanosomiasis.

The putative vectors of *L. infantum* in Israel are the sand fly species *P. perfiliewi*, *P. syricaus*, and *P. tobbi* [2, 3]. These species favor temperate climates with abundant vegetation, as found in the Mediterranean climate region of northern and central Israel, over the dry and hot climate present in the arid desert areas of southern Israel, where *P. papatasi*, the vector of *L. major*, is the dominant sand fly species [36]. The distribution of canine leishmaniosis cases in Israel found in the current study mostly parallels the distribution of the putative sand fly vectors of *L. infantum* in Israel. The majority of infected dogs were found in the northern and central part of Israel, and until 2019, the disease was detected only in dogs in these areas, whereas from 2020 to 2023, some cases have also been detected from the south of Israel (Fig. 5). This trend of canine infection spreading southward from 2020 to 2023 is worrying and may also be relevant to the recent description of human cutaneous leishmaniasis caused by *L. infantum* in southern Israel [27].

The epidemiology of canine leishmaniosis in Israel is different from human leishmaniasis and presents a contrary picture. A retrospective study examining 4168 cases of human leishmaniasis in Israel between the years 2017 and 2022 showed that 84% of patients were infected with *L. major*, 14.4% with *L. tropica*, 0.95% with New World *Leishmania* spp. (acquired outside of Israel), and only 0.65% with *L. infantum*. Twenty-two of the *L. infantum* cases were cutaneous leishmaniasis patients, with only five typical visceral leishmaniasis affecting internal organs [37]. In contrast, canine leishmaniosis in Israel is mostly caused by *L. infantum* and presents as a viscero-cutaneous disease, whereas infection with *L. major* and *L. tropica* occurs, but is rare.

The limitations of this study included missing data for some of the dogs in the study, such as exact location, age, and sex. It should also be taken into account that dogs can be infected in areas different from where they were diagnosed owing to travel, adoption, or change of owner’s address. Furthermore, although dog registration is mandatory by law in Israel, not all dogs are registered. The percentage of unregistered dogs in the total populations is not known but is assumed to be relatively constant throughout the years. It should be taken into consideration that the true number of diseased dogs and the rate of underdiagnosis are not fully known. In addition, dogs diagnosed at veterinary clinics by cytology, serological kits, or histopathological techniques demonstrating *Leishmania* sp. parasites at laboratories other than the VBDR were not included in this study, and these missing data mask the true burden of the disease, leading to possible underestimation of its actual impact.

## Conclusions

This is the first study to summarize and report on the spread and prevalence of canine leishmaniasis in Israel, where three species of *Leishmania* infect dogs. The disease is present in northern and central Israel, and is currently encroaching on the southern part of the country. Despite the low number of human leishmaniasis cases due to *L. infantum*, the presence of infected dogs in densely populated areas throughout Israel constitutes a reservoir for parasite transmission to canines, cats, and humans. Therefore, human leishmaniasis can occur in any region where infected dogs are present in Israel.

## Supplementary Information

Additional file 1: Table 1. Number of dogs registered between 2013 and 2022 at the Israeli Ministry of Agriculture. Data were retrieved from the annual reports of the Israeli Veterinary Services [9]. Figure 1. The sex distribution and male–female ratio (black line) among cases from 2014 to 2023. Figure 2. Map of Israel showing a kernel density estimation (KDE) performed to assess the spatial distribution of reported canine leishmaniosis disease cases in Israel. Table 2. The *Leishmania* species identification of infected dogs included in the study between 2010 and 2023 for which there was PCR and DNA sequencing. The GenBank accession number is provided for those samples that were deposited in GenBank and the BLAST search results showing the closest GenBank accession.

## Data Availability

The data supporting the findings of the study must be available within the article and/or its supplementary materials.
