# Supplementary material for: Retrospective study of canine leishmaniosis in Israel
Source: Parasit Vectors. 2025 Jul 4;18:257. doi: 10.1186/s13071-025-06862-4 (PMC12228340; doi:10.1186/s13071-025-06862-4)
Supplement: Supplementary file 1 — Additional file 1: Table 1. Number of dogs registered between 2013 and 2022 at the Israeli Ministry of Agriculture. Data were retrieved from the annual reports of the Israeli Veterinary Services [9]. Figure 1. The sex distribution and male–female ratio (black line) among cases from 2014 to 2023. Figure 2. Map of Israel showing a kernel density estimation (KDE) performed to assess the spatial distribution of reported canine leishmaniosis disease cases in Israel. Table 2. The Leishmania species identification of infected dogs included in the study between 2010 and 2023 for which there was PCR and DNA sequencing. The GenBank accession number is provided for those samples that were deposited in GenBank and the BLAST search results showing the closest GenBank accession. [file 13071_2025_6862_MOESM1_ESM.doc]

**Additional files**

**Additional file 1: Additional Table 1**. Number of dogs registered between 2013-2022 at the Israeli Ministry of Agriculture. Data was retrieved from the annual reports of the Israeli Veterinary Services [9].

| **Year** | **Total number of registered dogs** | **Males** | **Females** | **No sex registered** | **Ratio between males to females** |
| --- | --- | --- | --- | --- | --- |
| **2007** | 297,095 | 145,390 | 143,043 | 8,662 | 1.02 |
| **2008** | 346,878 | 173,981 | 170,557 | 2,340 | 1.02 |
| **2009** | **386,433** | **194,431** | **190,748** | 1,254 | 1.02 |
| **2010** | **387,289** | **195,592** | **191,697** | 0 | 1.02 |
| **2011** | **385,163** | **192,937** | **191,552** | 674 | 1.01 |
| **2012** | **411,123** | **208,338** | **202,369** | 416 | 1.03 |
| **2013** | **354,028** | **174,450** | **179,578** | 0 | 0.97 |
| **2014** | **383,181** | **198,209** | **184,972** | 0 | 1.07 |
| **2015** | 3**97,931** | **210,530** | **187,401** | 0 | 1.12 |
| **2016** | **444,395** | **225,679** | **218,716** | 0 | 1.03 |
| **2017** | **445,80**8 | **225,715** | **220,092** | 0 | 1.03 |
| **2018** | **477,088** | **240,933** | **235,929** | 0 | 1.02 |
| **2019** | **496,538** | **251,291** | **245,247** | 0 | **1.02** |
| **2020** | **527,647** | **267,765** | **259,683** | 199 | **1.03** |
| **2021** | **567,361** | **289,347** | **278,014** | 0 | **1.04** |
| **2022** | **581,052** | **296,562** | **284,460** | 0 | **1.04** |
| **2023** | **604,874** | **309,292** | **295,582** | 0 | 1.05 |

**Additional file 2: Additional Figure 1**. The sex distribution and male-female ratio (black line) among cases from 2014-2023.


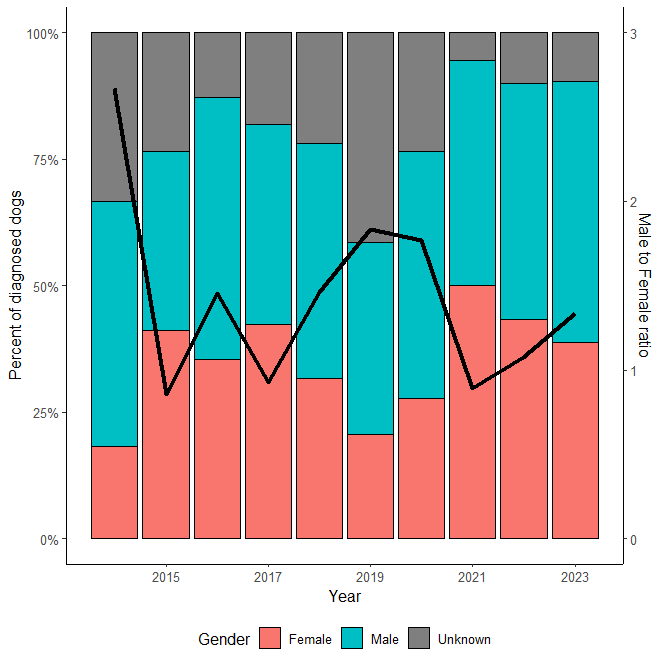


**Additional file 3: Additional Figure 2**. Map of Israel showing a kernel density estimation (KDE) performed to assess the spatial distribution of reported canine leishmaniosis disease cases in Israel.


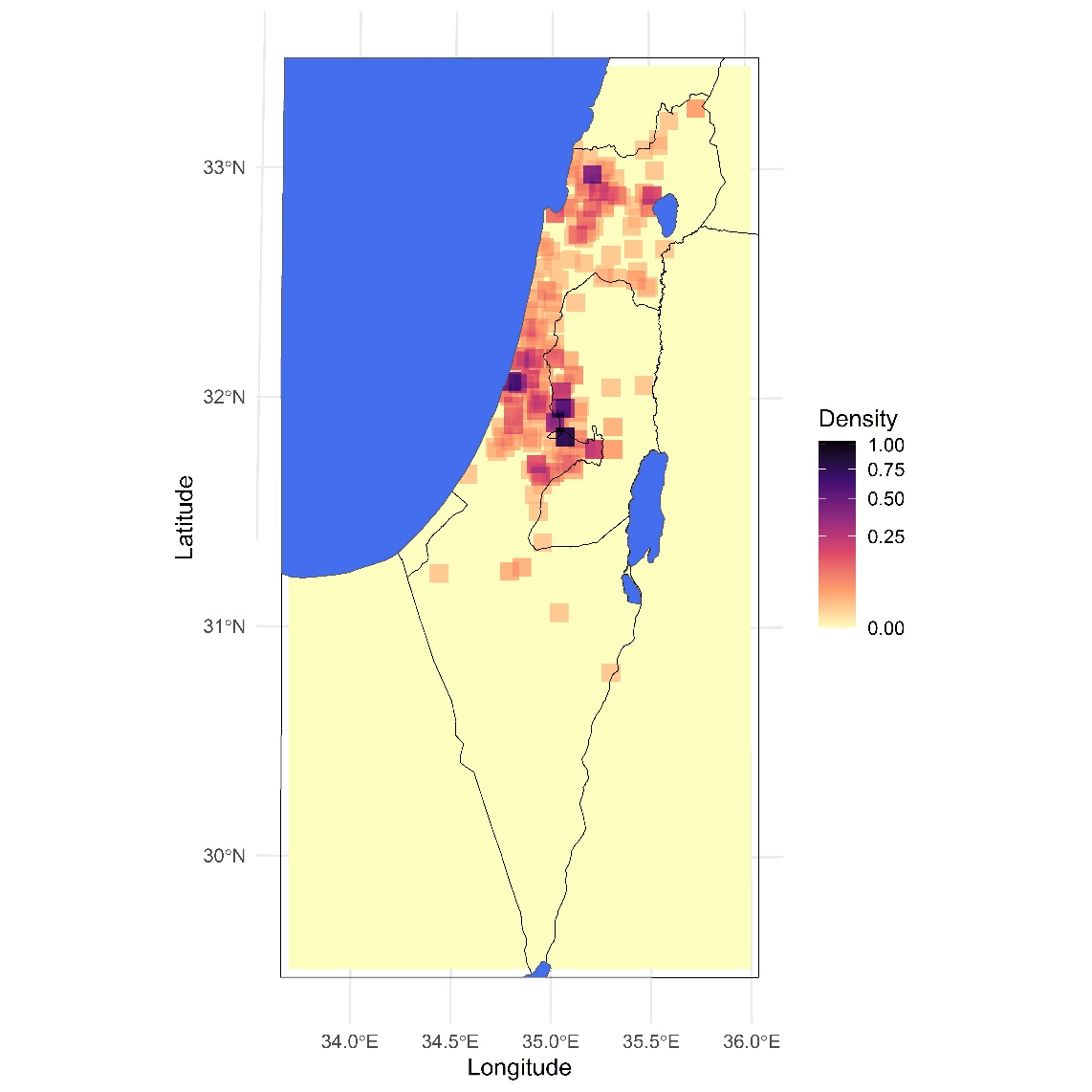


**Additional file 4: Additional Table 2**. The *Leishmania* species identification of infected dogs included in the study between 2010-2023 for which there was PCR and DNA sequencing. The GenBank accession number is provided for those samples which were deposited in GenBank and the BLAST search results showing the closest GenBank accession. GenBank accessions of dogs from this study which have been submitted prior to this study are in bold letters.

| Dog sample number | Year of diagnosis | *Leishmania* spp. | GenBank Accession number | BLASTn results (closest GenBank accession; % identity; % cover) |
| --- | --- | --- | --- | --- |
| 268 | 2010 | *L. infantum* | **KM677130** | *Leishmania infantum* (MG778644.1, 100%,100%) |
| 279 | 2011 | *L. infantum* | **KM677144** | *Leishmania infantum* (OR157993.1, 100%,100%) |
| 282 | 2011 | *L. infantum* | **KM677135** | *Leishmania infantum* (OR157993.1, 100%,100%) |
| 257 | 2011 | *L. infantum* | **KM677128** | *Leishmania infantum* (OR157993.1, 100%,100%) |
| 284 | 2011 | *L. infantum* | **KM677137** | *Leishmania infantum* (OR157993.1, 100%,100%) |
| 285 | 2011 | *L. infantum* | **KM677136** | *Leishmania infantum* (OM033717., 100%,100%) |
| 276 | 2011 | *L. infantum* | **KM677129** | *Leishmania infantum* (MG778644.1, 100%,100%) |
| 264 | 2012 | *L. infantum* | **KM677131** | *Leishmania infantum* (MG778644.1, 100%,100%) |
| 278 | 2011 | *L. infantum* | **KM677132** | *Leishmania infantum* (OR157993.1, 100%,100%) |
| 9910 | 2013 | *L. tropica* | **KF974365** | *Leishmania tropica* (FJ948464.1, 99%,100%) |
| 8647 | 2015 | *L. major* | **KU949582** | *Leishmania major* (MZ502964.1, 100%,100%) |
| 384 | 2015 | *L. tropica* | **KY524300** | *Leishmania tropica* (FJ948456.1, 100%,100%) |
| 8845 | 2016 | *L. major* | **KY524299** | *Leishmania major* (KP773413.1, 100%,100%) |
| 9136 | 2018 | *L. tropica* | **PV658138** | *Leishmania tropica* (KJ420584.1, 100%,100%) |
| 9295 | 2019 | *L. infantum* | PV658139 | *Leishmania infantum* (MF977314.1, 100%,100%) |
| 9296 | 2019 | *L. infantum* | PV658140 | *Leishmania infantum* (MF977314.1, 100%,100%) |
| 9299 | 2019 | *L. infantum* | PV658141 | *Leishmania infantum* (MF977314.1, 100%,100%) |
| 9315 | 2019 | *L. infantum* | PV658143 | *Leishmania infantum* (MF977314.1, 100%,100%) |
| 9328 | 2019 | *L. infantum* | PV658142 | *Leishmania infantum* (MF977314.1, 100%,100%) |
| 2064412 | 2020 | *L. infantum* | PV658144 | *Leishmania infantum* (MN503527.1, 100%,100%) |
| 2101857 | 2021 | *L. tropica* | PV658145 | *Leishmania tropica* (KJ420584.1, 98.5%,99%) |
| 2159734 (9387) | 2021 | *L. infantum* | PV658146 | *Leishmania infantum* (MF977314.1, 100%,100%) |
| 2160880 | 2021 | *L. infantum* | PV658147 | *Leishmania infantum* (OP724555.1, 99.5%, 100%) |
| 2359822 | 2023 | *L. infantum* | PV658148 | *Leishmania infantum* (MN503527.1, 100%,100%) |
| 8710 | 2015 | *L. infantum* | PV658149 | *Leishmania infantum* (MF977314.1, 100%,100%) |
| 8714 | 2015 | *L. infantum* | PV658150 | *Leishmania infantum* (MF977314.1, 100%,100%) |
| 8793 | 2016 | *L. infantum* | PV658151 | *Leishmania infantum* (MF977314.1, 100%,100%) |
| 8757 | 2016 | *L. infantum* | PV658152 | *Leishmania infantum* (MF977314.1, 100%,100%) |
| 8799 | 2016 | *L. infantum* | PV658153 | *Leishmania infantum* (MF977314.1, 100%,100%) |
| 8818 | 2016 | *L. infantum* | PV658154 | *Leishmania infantum* (MF977314.1, 100%,100%) |
| 8848 | 2016 | *L. infantum* | PV658155 | *Leishmania infantum* (MF977314.1, 100%,100%) |
| 8632 | 2014 | *L. infantum* | PV658156 | *Leishmania infantum* (MF977314.1, 100%,100%) |
